# Prediction of adolescent suicidal ideation after the COVID-19 pandemic: A nationwide survey of a representative sample of Korea

**DOI:** 10.3389/fped.2022.951439

**Published:** 2022-07-25

**Authors:** Haewon Byeon

**Affiliations:** ^1^Department of Digital Anti-Aging Healthcare (BK21), Graduate School, Inje University, Gimhae, South Korea; ^2^Department of Medical Big Data, College of AI Convergence, Inje University, Gimhae, South Korea

**Keywords:** suicidal ideation, COVID-19 pandemic, XGBoost, machine learning, subjective stress

## Abstract

**Objective:**

This study developed a model to predict groups vulnerable to suicidal ideation after the declaration of the COVID-19 pandemic based on nomogram techniques targeting 54,948 adolescents who participated in a national survey in South Korea.

**Methods:**

This study developed a model to predict suicidal ideation by using logistic regression analysis. The model aimed to understand the relationship between predictors associated with the suicidal ideation of South Korean adolescents by using the top seven variables with the highest feature importance confirmed in XGBoost (extreme gradient boosting). The regression model was developed using a nomogram so that medical workers could easily interpret the probability of suicidal ideation and identify groups vulnerable to suicidal ideation.

**Results:**

This epidemiological study predicted that eighth graders who experienced depression in the past 12 months, had a lot of subjective stress, frequently felt lonely in the last 12 months, experienced much-worsened household economic status during the COVID-19 pandemic, and had poor academic performance were vulnerable to suicidal ideation. The results of 10-fold cross-validation revealed that the area under the curve (AUC) of the adolescent suicidal ideation prediction nomogram was 0.86, general accuracy was 0.89, precision was 0.87, recall was 0.89, and the F1-score was 0.88.

**Conclusion:**

It is required to recognize the seriousness of adolescent suicide and mental health after the onset of the COVID-19 pandemic and prepare a customized support system that considers the characteristics of persons at risk of suicide at the school or community level.

## Introduction

Since the WHO declared the COVID-19 pandemic in March 2020, the world has been experiencing a crisis of COVID-19 until now. The COVID-19 pandemic has affected all age groups. Especially, the youth have experienced indirect effects such as the unemployment of their parents and the separation from society or peers due to the school lockdown, as well as direct effects such as infection. In 2020, as COVID-19 spread in South Korea, the government extended the winter break of elementary, middle, and high schools nationwide three times, and schools started on April 6 instead of March 2. Moreover, as the social distance step was raised to the lockdown level, intensive COVID-19 response measures were implemented. For example, face-to-face classes were banned and they were replaced with online classes (1).

The UN (2020) was concerned that the crisis of the COVID-19 pandemic, an infectious disease, was rapidly increasing a crisis in children’s health. Moreover, Choi (2) named the youth group suffering from the COVID-19 pandemic as the “COVID generation” and recommended showing more attention to their mental health because the COVID generation experienced interactions such as interpersonal relationships much less than the generations before the COVID-19 pandemic. People are highly concerned about its effect on suicidality after the outbreak of the COVID-19 pandemic. COVID-19 may increase the risk of developing suicidal behaviors by affecting numerous well-established suicide risk factors (3–5). Nevertheless, there are still not enough epidemiological studies using big data to identify groups vulnerable to mental health deterioration among adolescents after the COVID-19 pandemic.

In contrast, suicide refers to the act of taking own life with the intention of causing death (6). Adolescence is a stormy period, a transition period from childhood to adulthood, and adolescents may resolve the confusion about their identity and the uncertainty about their future by using extreme methods such as suicide without achieving psychological stability and balance (7). Many researchers (8, 9) have emphasized the importance of early detection of adolescent suicide while pointing out that adolescents with dangerous levels of suicidal ideation may not receive help early enough because suicidal ideation in adolescence is recognized by those around them as a universal psychological state that can appear during development. It was reported that the suicide rate of South Korea was the highest among OECD member countries as of 2020 (6). Especially, the suicide rate of teenagers skyrocketed by 9.4% compared to 2019 (10) before the COVID-19 pandemic. The result implied that youth suicide became a serious problem in South Korea after the outbreak of COVID-19.

Most previous studies (11–13) that identified predictors of suicidal ideation in adolescents mainly used logistic regression models. The logistic regression model is a stochastic model widely used to predict the likelihood of an event by linearly combining independent variables when the dependent variable is binomial. Although logistic regression analysis has the advantage of being able to identify the influence of individual variables on a dependent variable, it has a limitation in understanding the interaction between various explanatory variables used for the predictive model because it assumes independence that the effect of an explanatory variable does not depend on the level of other explanatory variables (14). As a way to overcome these limitations of regression analysis, many recent studies (15, 16) are widely using boosting-based machine learning models such as XGBoost (extreme gradient boosting).

Since the COVID-19 pandemic has not yet come to an end even after suffering from it for 2 years, it is necessary to conduct more studies based on scientific evidence to improve the mental health of the youth and prevent suicide. This study developed a model to predict groups vulnerable to suicidal ideation after the declaration of the COVID-19 pandemic based on nomogram techniques targeting 54,948 adolescents who participated in a national survey in South Korea.

## Materials and methods

### Data source

It was a secondary data analysis study using the 2020 Korea Youth Risk Behavior Survey (17). The Korea Youth Risk Behavior Survey is an anonymous self-reporting online survey targeting students between 7th-grade students and 12th-grade students to understand the health behaviors of South Korean adolescents. It was jointly conducted by the Ministry of Education, the Ministry of Health and Welfare, and the Korea Disease Control and Prevention Agency in South Korea. This study sampled subjects from the 2020 Korea Youth Risk Behavior Survey in the steps of population stratification, sample allocation, and sampling. In the sample allocation step, this study set the sample size to 400 middle schools and 400 high schools. Then, five middle schools and five high schools were first allocated to each of 17 cities and provinces. This study investigated 57,925 students who were selected as samples from 1 August 2020 to 30 November 2020. The participation rate was 94.9% (54,948 students). This study excluded students who were absent for more than 3 months, students with disabilities (e.g., intellectual disability), and students with dyslexia at the time of the investigation. The data collection method was an anonymous self-report online survey, if there were any unanswered items, it did not move on to the next item. Therefore, there was no missing value. All data were collected in a way that did not reveal personal identifiable information. This study analyzed the data of 54,948 subjects who responded that they had suicidal ideation among students between 7th grade and 12th grade who participated in the 2020 Korea Youth Risk Behavior Survey.

### Measurement of variables

The presence of suicidal ideation, an outcome variable, was determined when a subject responded “yes” to the item, “Have you ever seriously considered committing suicide in the past 12 months?”

The input variables included grade (1 = 7th grade, 2 = 8th grade, 3 = 9th grade, 4 = 10th grade, 5 = 11th grade, or 6 = 12th grade), gender (1 = male or 2 = female), subjective economic status (1 = high, 2 = medium, or 3 = low), whether the economic status has changed during the COVID-19 pandemic (1 = strongly agree, 2 = agree, 3 = disagree, or 4 = strongly disagree), living with a family member (1 = yes or 2 = no), area of residence (1 = urban area or 2 = rural area), school type (1 = middle school, 2 = vocational high school, or 3 = general high school), academic performance (1 = high, 2 = medium-high, 3 = medium, 4 = medium-low, or 5 = low), drinking at least one glass or shot of beer, soju, or whiskey within the last 30 days (1 = no or 2 = yes), smoking at least one cigarette within the last 30 days (1 = no or 2 = yes), drug experience (e.g., hallucinogens and drugs such as methamphetamine) (1 = no or 2 = yes), conflict in relationship with friends or colleagues due to smartphone overdependence (1 = strongly disagree, 2 = disagree, 3 = agree, or 4 = strongly agree), days of conducting moderate- or higher-intensity exercise regularly (none, 1–2 times a week, or 3 or more times a week), subjective sleep satisfaction (1 = sufficient, 2 = moderate, or 3 = insufficient), subjective health recognition (1 = good, 2 = moderate, or 3 = bad), subjective stress recognition (1 = high, 2 = moderate, or 3 = none), subjective body type recognition (1 = underweight, 2 = moderate, or 3 = obesity), weight control efforts in the past 30 days (1 = no effort or 2 = effort), sexual relation (1 = no or 2 = yes), depression experience in the past 12 months (1 = no or 2 = yes), experience of loneliness in the past 12 months (1 = rarely, 2 = moderate, or 3 = frequent), and receiving treatment due to violence from an acquaintance (e.g., adult, senior, or friend) (1 = no or 2 = yes). Depression was defined as the case of answering “yes” to “Have you ever felt sad or hopeless enough to stop your daily activities for 2 weeks in the past 12 months?” that was a criterion for determining major depressive disorder. Smartphone overuse was defined as the experience of severe conflict in a friend, colleague, or social relation due to smartphone overuse in the past 30 days. Regular moderate- or higher-intensity exercise was defined as “the experience of conducting exercise (regardless of exercise type) at the intensity that increases heart rate than usual or makes you out of breath for 60 minutes or longer in total per day in the past seven days.” Subjective sleep satisfaction was defined as the case in which the amount of sleep was sufficient to overcome fatigue in the last 7 days.

### Variable selection

A nomogram generally identifies the predictive path of disease by using 5–7 variables because when a larger number of explanatory variables were entered into the nomogram, the number of cases for calculating the predictive probability for a disease increases as well (18). Therefore, when developing a nomogram, it is important to select explanatory variables to be used in the nomogram. This study used XGBoost to select variables, and the top seven variables with high-feature importance were selected as variables to be used in the nomogram. XGBoost is a boosting technique that has the advantages of fast speed and scalability (19). XGBoost is based on a decision tree-based algorithm that uses a boosting technique that lowers the error by coupling multiple classification and regression trees. XGBoost generates an optimized model in a way that controls the complexity of the tree to minimize training loss and prevent overfitting. The objective function of XGBoost (19) is presented in the following equation:


(1) $$\text{obj}\,\left(\theta \right)=\sum_{i}^{n}l\,\left(y_{i},{\hat{y}}_{i}\right)+\sum_{j}^{k}\Omega \,\left(f_{k}\right)$$

where *K* stands for the number of trees, and Ω refers to all situations that may affect the complexity of trees. Starting from a tree with a depth of 0, if a lot of new information is gained (Gain) when pruning, the tree continues to grow (greedy learning of the tree) (20). The gain function of XGBoost is presented in the following equation:


(2) $$\text{Gain}=\frac{1}{2}\,\left[\frac{G_{L}^{2}}{H_{L}+\lambda }+\frac{G_{R}^{2}}{H_{R}+\lambda }-\frac{{\left(G_{L}+G_{R}\right)}^{2}}{H_{L}+H_{R}+\lambda }\right]-\gamma$$

Although XGBoost has been mainly used as a predictive model, it can also be used as an interpretable model for variable selection. This is because it is possible to understand the accuracy contribution score (gain) of each variable and the appearance frequency of the variable in the entire tree until the XGBoost model is formed by checking the feature importance (20). It can also confirm the split used for each pruning and the gain due to the split. It helps to understand the direction of the variable. This study set the hyperparameters of XGBoost as the number of trees = 100, learning rate = 0.3, regularization lambda = 1, and limit the depth of individual tree = 6.

### Development and validation of the nomogram

This study developed a model to predict suicidal ideation by using logistic regression analysis. The model aimed to understand the relationship between predictors associated with the suicidal ideation of South Korean adolescents by using the top seven variables with the highest feature importance confirmed in XGBoost. The regression model analyzed using multiple logistic regression with adjusted confounding factors. It presented an adjusted odds ratio (AOR) and 95% confidence interval (CI) to understand the independent relationship between predictive factors and adolescent suicidal ideation.

The regression model was developed using a nomogram so that medical workers could easily interpret the probability of suicidal ideation and identify groups vulnerable to suicidal ideation. The nomogram based on logistic regression is a two-dimensional diagram presenting the relationship between multiple risk factors to simply and efficiently calculate the predictive probability of disease (21). A logistic regression nomogram is generally composed of a point line, a risk factor line, a probability line, and a total point line (22). The point line is placed at the top of the nomogram to derive a score corresponding to the class of each risk factor (23, 24). Moreover, the number of risk factor line is equal to the number of risk factors for adolescent suicidal ideation. This study set the number of risk factor lines as seven for efficient interpretation of the nomogram. The total point line refers to the sum of the scores of individual risk factors. The probability line is the final risk probability value calculated based on the total point line and is placed at the bottom of the nomogram.

## Results

### General characteristics of subjects by suicidal ideation experience after the COVID-19 pandemic

Table 1 shows the differences (Chi-square test results) in the general characteristics between adolescents who experienced suicidal ideation during the COVID-19 pandemic and those who did not experience suicidal ideation during the COVID-19 pandemic. Among 54,948 South Korean adolescents, 5,979 adolescents (10.9%) experienced suicidal ideation during the COVID-19 pandemic. The results of Chi-square test showed that adolescents who experienced suicidal ideation and those who did not experience suicidal ideation were significantly different in grade, gender, whether the economic status has changed during the COVID-19 pandemic, household economic status, living with a family member, school type, area of residence, academic performance, drinking within the last 30 days, smoking within the last 30 days, conflict in social relationships due to smartphone overdependence, habitual drug use, regular physical activity, subjective sleep satisfaction, subjective health recognition, subjective stress recognition, subjective body-type recognition, weight control efforts in the past 30 days, sexual relation, depression experience in the past 12 months, receiving treatment due to violence from an acquaintance, and experience of loneliness in the past 12 months (*p* < 0.05).

**TABLE 1:** General characteristics of subjects according to suicidal ideation, *n* (%).

Variables	Suicidal ideation		*P*
	
	No (*n* = 48,969)	Yes (*n* = 5,979)	
Grade			<0.001
7th grade	9,108 (91.0)	897 (9.0)	
8th grade	8,501 (88.9)	1,063 (11.1)	
9th grade	8,339 (88.8)	1,053 (11.2)	
10th grade	7,981 (89.6)	926 (10.4)	
11th grade	7,822 (87.8)	1,085 (12.2)	
12th grade	7,218 (88.3)	955 (11.7)	
Gender			<0.001
Male	26,099 (92.1)	2,254 (7.9)	
Female	22,870 (86.0)	3,725 (14.0)	
Changes in economic status due to COVID-19			<0.001
Strongly agree	2,637 (81.0)	619 (19.0)	
Agree	11,817 (87.0)	1,766 (13.0)	
Disagree	19,641 (89.9)	2,200 (10.1)	
Strongly disagree	14,874 (91.4)	1,394 (8.6)	
Household economic status			<0.001
High	19,331 (90.6)	2,008 (9.4)	
Moderate	23,758 (90.0)	2,639 (10.0)	
Low	5,880 (81.5)	1,332 (18.5)	
Living with a family member			<0.001
Yes	46,738 (89.3)	5,594 (10.7)	
No	2,231 (85.3)	385 (14.7)	
School type			<0.001
Middle school	25,948 (89.6)	3,013 (10.4)	
Vocational high school	4,462 (89.7)	514 (10.3)	
General high school	18,559 (88.3)	2,452 (11.7)	
Area of residence			<0.001
Urban	21,181 (89.7)	2,440 (10.3)	
Rural	27,788 (88.7)	3,539 (11.3)	
Academic performance			<0.001
High	6,081 (90.3)	655 (9.7)	
Medium-high	12,123 (90.4)	1,278 (9.6)	
Medium	1,5034 (90.6)	1,551 (9.4)	
Medium-low	11,150 (87.9)	1,534 (12.1)	
Low	4,581 (82.8)	952 (17.2)	
Drinking within the last 30 days			<0.001
No	44,247 (90.2)	4,809 (9.8)	
Yes	4,722 (80.1)	1,170 (19.9)	
Smoking within the last 30 days			<0.001
No	47,311 (89.6)	5,499 (10.4)	
Yes	1,658 (77.5)	480 (22.5)	
Conflict in social relation due to smartphone overdependence			<0.001
Strongly disagree	33,257 (90.6)	3,454 (9.4)	
Disagree	14,065 (88.0)	1,924 (12.0)	
Agree	1,338 (75.1)	444 (24.9)	
Strongly agree	309 (66.3)	157 (33.7)	
Habitual drug use			<0.001
No	48,738 (89.4)	5,805 (10.6)	
Yes	231 (57.0)	174 (43.0)	
Regular physical activity			0.009
No	18,720 (88.7)	2,391 (11.3)	
1–2 times a week	14,376 (89.1)	1,754 (10.9)	
3 or more times a week	15,873 (89.6)	1,834 (10.4)	
Subjective sleep satisfaction			<0.001
Sufficient	15,850 (94.2)	974 (5.8)	
Moderate	16,868 (90.4)	1,788 (9.6)	
Insufficient	16,251 (83.5)	3,217 (16.5)	
Subjective health recognition			<0.001
Good	35,395 (92.1)	3,049 (7.9)	
Moderate	10,543 (85.4)	1,799 (14.6)	
Bad	3,031 (72.8)	1,131 (27.2)	
Subjective stress recognition			<0.001
High	14,208 (76.1)	4,454 (23.9)	
Moderate	23,055 (94.6)	1,324 (5.4)	
None	11,706 (98.3)	201 (1.7)	
Subjective body type recognition			<0.001
Underweight	11,934 (89.6)	1,390 (10.4)	
Moderate	18,024 (90.8)	1,825 (9.2)	
Obesity	19,011 (87.3)	2,764 (12.7)	
Weight control efforts in the past 30 days			<0.001
No effort	22,419 (90.5)	2,355 (9.5)	
Effort	26,550 (88.0)	3,624 (12.0)	
Sexual relation			<0.001
No	47,050 (89.7)	5,411 (10.3)	
Yes	1,919 (77.2)	568 (22.8)	
Depression experience in the past 12 months			<0.001
No	39,468 (96.0)	1,640 (4.0)	
Yes	9,501 (68.6)	4,339 (31.4)	
Receiving treatment due to violence from an acquaintance			<0.001
No	48,530 (89.5)	5,699 (10.5)	
Yes	439 (51.1)	280 (38.9)	
Experience of loneliness in the past 12 months			<0.001
Rarely	27,358 (96.8)	901 (3.2)	
Moderate	16,723 (88.4)	2,200 (11.6)	
Frequently	4,888 (62.9)	2,878 (37.1)	

### Predictive factors for suicidal ideation in South Korean adolescents

**FIGURE 1:**
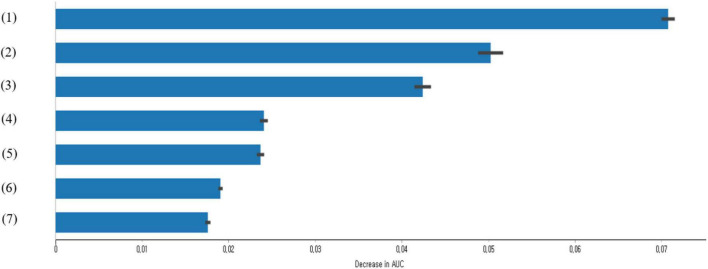
The features importance of factors related to the suicidal ideation of adolescents using XGBoost (only top seven factors are presented): (1) depression experience in the past 12 months; (2) subjective stress recognition; (3) experience of loneliness in the past 12 months; (4) academic performance; (5) grade; (6) household economic status; and (7) changes in economic status due to COVID-19.

This study calculated the feature importance of factors associated with the suicidal ideation of South Korean adolescents by using XGBoost (Figure 1). The results showed that the top seven variables with high-feature importance were depression experience in the past 12 months, subjective stress recognition, experience of loneliness in the past 12 months, academic performance, grade, household economic status, and changes in economic status due to COVID-19.

Table 2 shows the results of logistic regression analysis for predicting the suicidal ideation of South Korean adolescents using the top seven variables with high-feature importance in XGBoost. The analysis results of adjusted model for predicting the suicidal ideation of South Korean adolescents showed that independent influencing factors were 7th grade (AOR = 1.15, 95% CI = 1.03–1.28), 12th grade (AOR = 0.89, 95% CI = 0.80–0.99), adolescents with very large economic changes due to COVID-19 (AOR = 1.25, 95% CI = 1.10–1.41), adolescents with poor household economic status (AOR = 1.26, 95% CI = 1.14–1.38), adolescents with moderate academic performance (AOR = 0.86, 95% CI = 0.77–0.96), adolescents who frequently experienced subjective stress (moderate: AOR = 1.88, high: AOR = 4.81), adolescents who experienced depression in the last 12 months (AOR = 4.85, 95% CI = 4.53–5.19), and adolescents who frequently experienced loneliness in the past 12 months (moderate: AOR = 1.93, frequently: AOR = 4.58) (*p* < 0.05).

**TABLE 2:** Predictors for suicidal ideation in South Korean adolescents: AOR and 95% CI.

Variables	AOR	95% CI	*P*
**Grade**			
7th grade (reference)	1	1	
8th grade	1.15	1.03, 1.28	0.008
9th grade	1.04	0.93, 1.16	0.437
10th grade	0.91	0.81, 1.01	0.103
11th grade	1.01	0.90, 1.12	0.893
12th grade	0.89	0.80, 0.99	0.044
**Changes in economic status due to COVID-19**			
Strongly agree	1.25	1.10, 1.41	<0.001
Agree	1.09	0.99, 1.19	0.056
Disagree	1.03	0.95, 1.12	0.396
Strongly disagree (reference)	1	1	
**Household economic status**			
High (reference)	1	1	
Moderate	0.96	0.89, 1.03	0.310
Low	1.26	1.14, 1.38	<0.001
**Academic performance**			
High (reference)	1	1	
Medium-high	0.93	0.83, 1.04	0.226
Medium	0.86	0.77, 0.96	0.009
Medium-low	0.92	0.82, 1.03	0.187
Low	1.09	0.96, 1.24	0.156
**Subjective stress recognition**			
High	4.81	4.13, 5.60	<0.001
Moderate	1.88	1.60, 2.19	<0.001
None (reference)	1	1	
**Depression experience in the past 12 months**			
No (reference)	1	1	
Yes	4.85	4.53, 5.19	<0.001
**Experience of loneliness in the past 12 months**			
Rarely (reference)	1	1	
Moderate	1.93	1.77, 2.11	<0.001
Frequently	4.58	4.18, 5.02	<0.001

### Development and validation of a nomogram for high-risk groups for suicidal ideation in Korean adolescents

**FIGURE 2:**
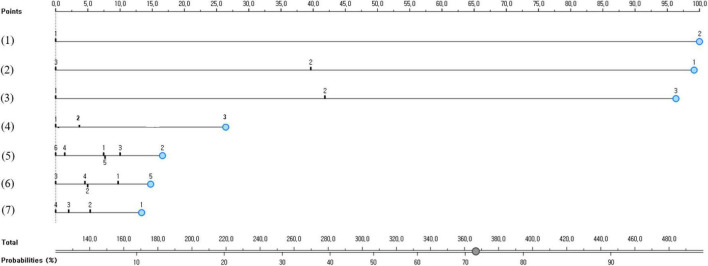
A nomogram predicting the South Korean adolescent group vulnerable to suicidal ideation: (1) depression experience in the past 12 months (1 = no or 2 = yes), (2) subjective stress recognition (1 = high, 2 = moderate, or 3 = none), (3) experience of loneliness in the last 12 months (1 = rarely, 2 = moderate, or 3 = frequent), (4) subjective economic status (1 = high, 2 = medium, or 3 = low), (5) grade (1 = 7th grade, 2 = 8th grade, 3 = 9th grade, 4 = 10th grade, 5 = 11th grade, or 6 = 12th grade), (6) academic performance (1 = high, 2 = medium-high, 3 = medium, 4 = medium-low, or 5 = low), and (7) changes in economic status during the COVID-19 pandemic (1 = strongly agree, 2 = agree, 3 = disagree, or 4 = strongly disagree).

The nomogram for predicting the suicidal ideation of South Korean adolescents is presented in Figure 2. This nomogram derived that the predictive probability of suicidal ideation for eighth graders who responded that they experienced depression in the past 12 months, they had a lot of subjective stress, they frequently felt lonely in the last 12 months, their household economic status worsened a lot during the COVID-19 pandemic, and their academic performance was very poor was 72%.

**FIGURE 3:**
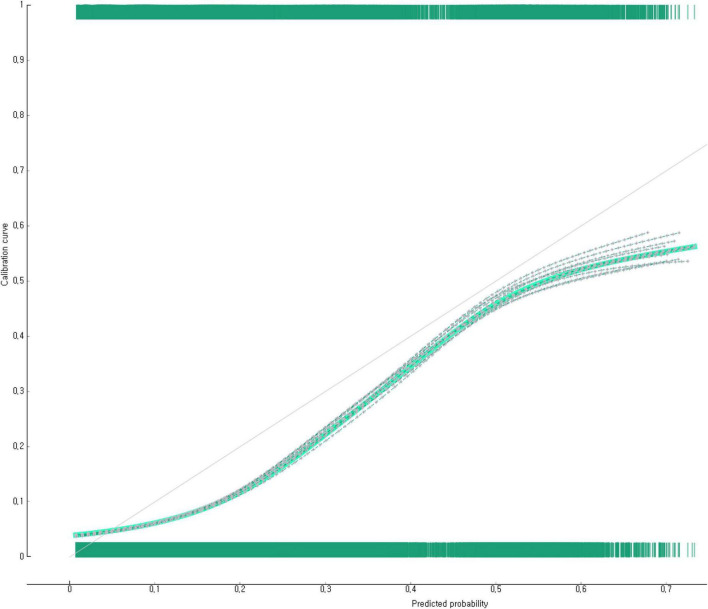
A calibration plot to identify the performance of the nomogram to predict the South Korean adolescent group vulnerable to suicidal ideation.

The predictive performance of the developed nomogram for predicting adolescent suicidal ideation was tested using AUC, general accuracy, F1, recall, precision, and calibration plot (Figure 3). The prediction probability and observation probability of the adolescent group that experienced suicidal ideation and those of the adolescent group that did not experience suicidal ideation were compared using calibration plot and Chi-square test (Figure 3). The results showed that prediction probability and observation probability were not significantly (*p* < 0.05) different. The results of 10-fold cross-validation revealed that the AUC of the adolescent suicidal ideation prediction nomogram was 0.86, the general accuracy was 0.89, the precision was 0.87, the recall 0.89, and the F1-score was 0.88.

## Discussion

This study evaluated the factors associated with the suicidal ideation of adolescents using epidemiological data representing South Korean adolescents. The results showed that middle-school students had a higher risk of suicidal ideation than high-school students. Previous studies (25, 26) on South Korean adolescents reported that middle-school students had a 1.3-fold higher risk of suicide attempts than high-school students, which agreed with the results of this study. Brière et al. (26) showed that eighth and ninth graders had the most suicidal ideation and suicide attempts. Glenn et al. (25) also revealed that suicide ideation increased abruptly between 12- and 14-year-old adolescents. Suicidal ideation refers to a continuous interest, thoughts, and illusions about ending one’s own life (27). Middle-school students who were adolescents could be more easily stuck in a psychologically maladjusted state (28) than high-school students when they experienced stress or negative life events. When this psychological maladjustment state persists, it is highly likely to develop mental health problems (29).

Dubé et al. (4) conducted a meta-analysis using 54 studies (308,596 subjects), which examined suicide behaviors during the COVID-19 pandemic, to find that event rates (e.g., 10.81% for suicidal ideation and 4.68% for suicide attempts) increased from studies conducted prior to the pandemic. Nevertheless, it is noteworthy that most of the studies targeted adults. Since only a few epidemiological studies analyzed the suicidal ideation of adolescents after the COVID-19 pandemic, additional epidemiological studies are needed to understand the difference between adolescent suicidal ideation before and after the pandemic.

Suicide has been a direct cause of death in South Korea and the top cause of death among South Korean adolescents for 10 consecutive years (30). Social efforts are required to maintain adolescent mental health because it has been reported that adolescents who experienced suicidal ideation have a high risk of suicide even if they do not choose to commit suicide during adolescence and may experience severe depression due to social maladjustment (26). Therefore, it is highly required to detect middle-school students highly vulnerable to suicide as soon as possible and intervene with them continuously to reduce the suicide rate of adolescents in the future based on the results of this study. It is also needed to develop a suicide prevention program tailored to the sociodemographic characteristics of middle-school students.

In this study, adolescents who experienced loneliness in the last 12 months had a higher risk of suicidal ideation. It seems that the result is related to emotional anxiety due to the absence of a person to seek help from. Choi et al. (31) examined the suicidal ideation of South Korean middle-school students and reported that only 32.3% of South Korean adolescents had consulted with others or asked for help when they have difficulties. The results implied that two out of three middle-school students tried to solve problems on their own without the help of people around them when encountering difficulties. It is believed that middle-school students frequently feel lonely and give up asking for help from people around them. As a result, they feel helpless repeatedly, which ultimately leads to suicidal ideation.

The results of this study confirmed that the level of stress perceived by adolescents was significantly related to suicidal ideation. These results were similar to the results of previous studies (32, 33) showing that stress was a major risk factor for suicide in adolescence. Stress that is perceived as not controllable is highly likely to make people lose the meaning of their lives (32). Moreover, persistent stress is highly likely to intensify suicidal ideation (33). Therefore, medical personnel need to first understand the stress level perceived by the subject more than anything else to detect adolescents with a high risk of suicidal ideation.

Another finding of this study was that change in household economic status due to the COVID-19 pandemic was identified as a major risk factor for adolescent depression. As the lockdown caused by the COVID-19 pandemic continued, South Korean workers experienced income reduction and instability due to business regulations (34, 35). Moreover, many business owners had to close their businesses in extreme cases, in addition to income decrease (34, 35). The decrease in household income due to the extended COVID-19 pandemic threatened the survival of the family (35). This economic difficulty could become a bigger psychological problem for economically vulnerable groups such as older adults and adolescents than adults (36). For example, as the lockdown due to the COVID-19 pandemic continued, students could experience isolation due to school closures and the absence of psychological support providers (37). As they experienced a crisis in the household economy at the same time, their psychological and emotional problems could be further exacerbated. The results of this study showed that psychological problems such as the suicidal ideation of adolescents were significantly related to rapid changes in household economic status, such as unemployment or a decrease in income of workers due to the COVID-19 pandemic. These results implied that the government should respond to the unemployment and reduced income of workers due to the extended COVID-19 pandemic more sensitively. They also suggested that there would be a need to pay attention especially to the mental health of the children of households with sharply declining incomes continuously.

This study developed a logistic nomogram and identified multiple risk factors for adolescent suicidal ideation during the COVID-19 pandemic. This nomogram derived that the predictive probability of suicidal ideation for eighth graders who responded that they experienced depression in the past 12 months, they had a lot of subjective stress, they frequently felt lonely in the last 12 months, their household economic status worsened a lot during the COVID-19 pandemic, and their academic performance was very poor was 72%, which was high. In South Korea, the teenage suicide rate in 2020 increased by 9.4% from that in 2019 (10). As the prolonged COVID-19 pandemic has not ended as of May 2022, it may increase even further in the future. Therefore, it is necessary to screen depression for the high suicidal ideation risk group with all these multiple risk factors at the school or community level and to conduct community-centered monitoring continuously to prevent depression. It is also required to conduct additional studies on multiple risk factors for suicidal ideation among adolescents after the COVID-19 pandemic.

The strengths of this study were to identify the adolescent group vulnerable to depressive disorder based on multiple risk factors and present the basis for selecting the adolescent group vulnerable to suicide based on the results. This study had several limitations. First, this study did not investigate parental abuse or negligence. Second, the variables used in this epidemiological study were measurements based on self-report questionnaires. Future studies need to identify risk factors for adolescent suicidal ideation by integrating qualitative research methods such as in-depth interviews in addition to self-report questionnaires. Third, since the results of this study were based on a cross-sectional study, the results cannot be interpreted as a causal relationship. It is necessary to conduct longitudinal studies on adolescents vulnerable to suicidal ideation identified in this study.

## Conclusion

This epidemiological study predicted that eighth graders who experienced depression in the past 12 months, had a lot of subjective stress, frequently felt lonely in the last 12 months, experienced much-worsened household economic status during the COVID-19 pandemic, and had poor academic performance were vulnerable to suicidal ideation (a high suicide risk group). Therefore, it is necessary to continuously intervene (e.g., early detection of adolescents vulnerable to suicidal ideation and mental health management) with adolescents to prevent adolescent suicide. It is also required to recognize the seriousness of adolescent suicide and mental health after the onset of the COVID-19 pandemic and prepare a customized support system that considers the characteristics of persons at risk of suicide at the school or community level.

## Data availability statement

The datasets presented in this study can be found in online repositories. The names of the repository/repositories and accession number(s) can be found below: https://www.kdca.go.kr/yhs/.

## Ethics statement

This study was conducted according to the guidelines of the Declaration of Helsinki, and approved by the Institutional Review Board (or Ethics Committee) of Korea Disease Control and Prevention Agency (protocol code 117075 and date: 01-07-2021). Written informed consent to participate in this study was provided by the participants or their legal guardian/next of kin.

## Author contributions

HB was involved in study data interpretation, designed the manuscript, performed the statistical analysis, and assisted with writing the manuscript.
